# Rumen Inoculum Enhances Cathode Performance in Single-Chamber Air-Cathode Microbial Fuel Cells

**DOI:** 10.3390/ma15010379

**Published:** 2022-01-05

**Authors:** Ignacio T. Vargas, Natalia Tapia, John M. Regan

**Affiliations:** 1 Department of Hydraulic and Environmental Engineering, Pontificia Universidad Católica de Chile, Santiago 7820436, Chile; netapia@uc.cl; 2 Centro de Desarrollo Urbano Sustentable (CEDEUS), Santiago 7520246, Chile; 3 Department of Civil and Environmental Engineering, The Pennsylvania State University, University Park, PA 16801, USA; jmr41@psu.edu

**Keywords:** microbial fuel cells, rumen fluid, cathodic biofilm

## Abstract

During the last decade, bioprospecting for electrochemically active bacteria has included the search for new sources of inoculum for microbial fuel cells (MFCs). However, concerning power and current production, a *Geobacter*-dominated mixed microbial community derived from a wastewater inoculum remains the standard. On the other hand, cathode performance is still one of the main limitations for MFCs, and the enrichment of a beneficial cathodic biofilm emerges as an alternative to increase its performance. Glucose-fed air-cathode reactors inoculated with a rumen-fluid enrichment and wastewater showed higher power densities and soluble chemical oxygen demand (sCOD) removal (Pmax = 824.5 mWm^−2^; ΔsCOD = 96.1%) than reactors inoculated only with wastewater (Pmax = 634.1 mWm^−2^; ΔsCOD = 91.7%). Identical anode but different cathode potentials suggest that differences in performance were due to the cathode. Pyrosequencing analysis showed no significant differences between the anodic community structures derived from both inocula but increased relative abundances of *Azoarcus* and *Victivallis* species in the cathodic rumen enrichment. Results suggest that this rarely used inoculum for single-chamber MFCs contributed to cathodic biofilm improvements with no anodic biofilm effects.

## 1. Introduction

Power densities achieved from microbial fuel cell (MFC) systems have increased by several orders of magnitude during the last decade. Before 2010, most of the reported results did not exceed 0.1 Wm^−2^. However, in recent years, power densities greater than 1 Wm^−2^ are more frequently found [1,2]. This rapid development has been primarily due to improvements in physical and chemical factors (e.g., architecture, electrode materials, membranes, and buffering) [1,3]. As these physicochemical considerations are addressed, opportunities emerge to enhance MFC performance through biological factors. Some of the current areas of microbial research in MFCs include exploring new sources of electrochemically active bacteria (EAB) with distinct physiologies, characterizing complex community interactions, and understanding the electron transfer mechanisms that govern current generations [4,5].

Bioprospecting for EAB has been an important motivation for microbial research in MFCs. The search for new sources of EAB has included sludge from wastewater plants [6,7], sediments [8,9], rice paddy fields [10], forest soils [9,11], and rumen fluids [12,13,14,15,16]. Extreme environments have also been tested, taking inocula from salt marshes [17], saline microbial mats [9], salt lakes [18], tropical mangrove sediments [19], and acid mine drainage affected sites [20]. Bioprospecting studies have allowed the detection of a diversity of electrogenic microorganisms, which have been shown to generate high power densities when tested in pure cultures. However, some studies showed that mixed cultures have a better power performance [21,22]. This could be mainly due to the cooperation between different microorganisms, allowing for the decomposing of heterogeneous substrates or providing a beneficial environment for anaerobic EABs [23]. This enormous effort has focused on the effect of these new inoculum sources on the anodic microbial community and its performance, leaving less attention to the impact on the cathode.

The cathode is often the main limitation for power production, and the cost of platinized cathodes is prohibitive in large-scale applications. Hence, there has been considerable interest in developing alternative cathode materials such as activated carbon and electrochemically active cathodic biofilms to improve MFC performance. A single-chamber air-cathode MFC offers an efficient and scalable configuration [24]. However, this cathode configuration sustains the involuntary growth of a biofilm on the air-cathode that increases coulombic efficiency (CE) but decreases power production [25,26]. To address this limitation, the potential enrichment of a beneficial cathodic microbial community represents an alternative strategy to increase cathode performance in MFC systems.

Cow rumen fluid offers an opportunity to enrich diverse and unique microbial communities in MFC systems, with the potential of increasing substrate degradation efficiency and adding new EAB to enhance power production. The rumen of an adult dairy cow contains from 10^9^ to 10^11^ bacteria per liter of fluid and a virtually unknown microbial ecology. Nagaraja [27] estimated that less than 10% of the rumen microorganisms had been phylogenetically identified. The main function of this impressively rich microbiota is to digest plant polysaccharides and ferment the released sugars to produce volatile fatty acids, which are absorbed by the ruminant as a source of energy [27]. Both strict and facultative anaerobes have been observed as members of rumen consortia, and it is common to find syntrophic relationships in a ruminal biofilm, as bacteria that degrade intermediary metabolites produce a more efficient substrate utilization [14,27]. Previous MFC articles have reported on the use of rumen material as an inoculum. Rismani-Yazdi et al. [14] and Chung et al. [13] used H-type MFC reactors to demonstrate that rumen microorganisms can hydrolyze cellulose and produce electric energy.

Additionally, Rismani-Yazdi et al. observed that the addition of rumen inoculum to the anode chamber increased the microbial diversity. Using 16S rRNA gene analysis, the authors identified *Firmicutes* as the dominant phylum in the anodic biofilm and *Betaproteobacteria* as the dominant phylum in the suspended culture. Different groups have used diverse MFC configurations with rumen inocula to demonstrate that it is possible to obtain electricity from an aquatic plant rich in cellulose, hemicellulose, and lignin [16,28,29,30,31]. Previous studies have also evaluated the effect of different catholytes on MFCs inoculated with rumen [12], the isolation of methanogens to increase power production in mixed community MFCs [15], and how the supplementation of dried red pepper modify the fermentation of rumen microorganism, thereby improving reactor performance [32]. Thus, previous studies have focused on anodic reactions and electricity production rather than quantifying how rumen ecology affects cathodic biofilm composition and the subsequent improvements on MFC performance. In this article, for the first time, cow rumen fluid was used to study the enrichment of both anodic and cathodic microbial communities in single-chamber air-cathode MFC reactors. Following microbial community analysis, the electrochemical performance of each biofilm and their effect on the overall power performance were quantified.

## 2. Materials and Methods

### 2.1. MFC Construction and Operation

Single-chamber air-cathode MFCs were constructed using cube-type plexiglass reactors of 28 mL volume. Graphite fiber brushes 2.5 cm in diameter and 2.5 cm long (PANEX 33 160K, ZOLTEK, Nyergesújfalu, Hungary) were placed horizontally in the cube reactors and used as anodes. Brushes were heat treated in a muffle furnace at 450 °C for 30 min [33]. Cathodes contained 0.5 mg/cm^2^ platinum catalyst applied to the water-facing side of carbon cloth (Type B-1B, E-TEK, 3.8 cm diameter, 7.1 cm^2^ of exposed surface area), and four polytetrafluoroethylene (PTFE) diffusion layers applied to the air-facing side [34].

MFCs were inoculated with effluent from a primary clarifier at The Pennsylvania State University Wastewater Treatment Plant (PSU-WWTP), a frequently used inoculum source for MFC reactors [2], and an enriched cow rumen sample. The rumen sample was collected from a cow at the dairy complex of The Pennsylvania State University and enriched for two weeks in a flask using H_2(g)_ and fumarate as electron donor and acceptor, respectively. Under this condition, enrichment of lithotrophic bacteria capable of using fumarate as electron acceptor, such as *Geobacter* spp. [35], was expected.

Reactors were fed a medium containing 1 g/L of glucose, 50 mM phosphate buffer solution (PBS), and mineral (12.5 mL/L) and vitamin (5 mL/L) solutions [33]. Two conditions were tested in duplicate reactors: (1) MFCs inoculated with 14 mL of a mix (50:50 *v*/*v*) of enriched rumen fluid and wastewater (RU) and (2) MFCs inoculated using 14 mL of only wastewater (WW). Since the rumen fluid was pre-enriched using fumarate as electron acceptor, two additional rumen reactors were, for each batch-cycle, amended with 60 mg L^−1^ of fumarate as an alternative electron acceptor.

MFCs were operated for 60 days as fed-batch reactors in a temperature-controlled room (30 ± 1 °C). Voltage was measured every 10 min across an external resistor of 1 kΩ using a data acquisition system (2700; Keithley, Cleveland, OH, USA). Batch cycles were 3 to 4 days, with the end of each cycle defined when voltage dropped below 20 mV [36]. Current and power were normalized to the cathode projected surface area. Anode and cathode potentials were measured with a 7.5 cm long Ag/AgCl reference electrode (MF-2079, BASi, West Lafayette, IN, USA) inserted close to the cathode and the distal end of the brush anode. Power densities were calculated using the highest ten voltages for each batch cycle, and CE was calculated based on current produced and chemical oxygen demand (COD) consumed as previously described [34]. To obtain polarization data, the external resistance was varied from 10 Ω to 40 kΩ, allowing the systems to reach stable voltage at each resistance.

### 2.2. Analyses

Liquid samples were obtained from the MFCs at the end of each cycle, filtered with a syringe filter (0.2 μm Supor Membrane, PALL Life Science, New York, NY, USA), and analyzed for soluble COD (sCOD) using the colorimetric method (Cat 21258-15; HACH, Loveland, CO, USA). Headspace gas samples (250 μL) were collected in duplicate using a gas-tight syringe and analyzed by a gas chromatograph Model 8610 (SRI Instruments, Torrance, CA, USA) equipped with a thermal conductivity detector and a stainless-steel column (1.8 m × 1/8″) packed with Porapak Q (Alltech, Deerfield, IL, USA).

One of the duplicates of each condition was used for electrochemical analysis (as follows) the other reactors were saved for community analysis, avoiding possible effects of electrochemical tests on community structure. Potentiostatic electrochemical impedance spectroscopy (EIS) was performed on anodes and cathodes using a Reference 600 potentiostat (Gamry Instruments Inc., Warminster, PA, USA). MFCs were tested during the first 24 h of several cycles. Before each test, reactors were disconnected for 2 h to reach open circuit potential (OCP). A 7.5 cm long Ag/AgCl electrode (MF-2079, BASi, West Lafayette, IN, USA) was used as reference; anode and cathode were used as working and counter electrodes. Anodic EIS was performed at OCP (~0.7 V), with an AC potential of 10 mV rms, frequencies from 10^5^ to 0.004 Hz, at 10 points per decade of data acquisition [37]. Cathodic EIS was performed at DC potentials of 0.3 V, 0.1 V, and 0.005 V, with an AC potential of 10 mV rms, frequencies from 10^6^ to 0.01 Hz, at 10 points per decade of data acquisition. Impedance spectra were fitted with an equivalent circuit by χ^2^ minimization using the software Echem Analyst (Gamry Instrument Inc., Warminster, PA, USA).

Differences among power densities were evaluated with a one-way analysis of variance (ANOVA) and using Microsoft Excel data analysis (Microsoft 365, Version 2111, 2020r, Microsoft Corporation, Redmond, WA, USA).

### 2.3. Bacterial Community Analysis

DNA from anodes and cathodes was, as according to previous MFC studies, extracted using a PowerSoil DNA Isolation Kit (MO BIO Laboratories, Carlsbad, CA, USA) [38]. Pyrosequencing analysis was performed using a Roche/454 Life Science Genome Sequencer [39] by amplification and sequencing of the 16S rRNA gene using the following bacterial primers: 27F (5′-AGAGTTTGATCMTGGCTCAG-3′) and 907R (5′-CCCCGTCAATTCMTTTGAGTTT-3′). The classification was against the manually curated SILVA gold aligned genes [40] using the Bayesian sequence classifier in the mother software package [41]. Classifications with differences of less than 0.5% were not reported.

## 3. Results and Discussion

### 3.1. MFC Performance

**Figure 1:**
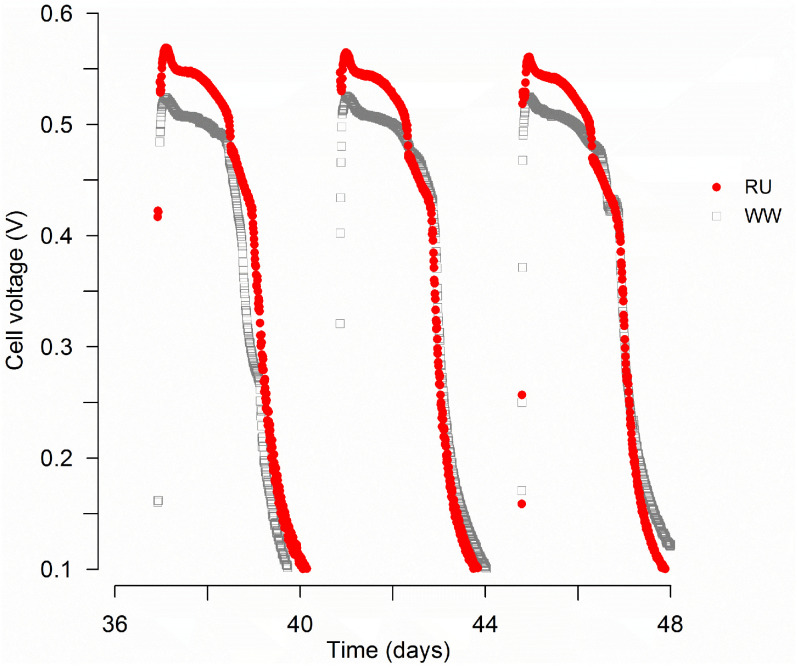
MFC reactors inoculated with a rumen enrichment showed higher cell voltages than reactors inoculated only with wastewater. RU: rumen plus wastewater; WW: wastewater. The figure shows the average results of duplicate reactors connected to external resistance of 1 kΩ over 3 batch cycles.

After 36 days of batch-cycle operation across an external resistance of 1 kΩ, stable voltage profiles were observed for all tested reactors (Figure 1).

MFCs inoculated with rumen fluid and wastewater showed 20% higher operating power densities (RU = 441 ± 10 mWm^−2^) than those inoculated only with wastewater (WW = 369 ± 28 mWm^−2^). Headspace gas measurements at the end of three consecutive batch cycles after 40 days of operation did not reveal significant differences in gas composition. While hydrogen gas was not detected for any condition, methane and carbon dioxide percentages were very similar for both treatments (Table 1).

**Figure 2:**
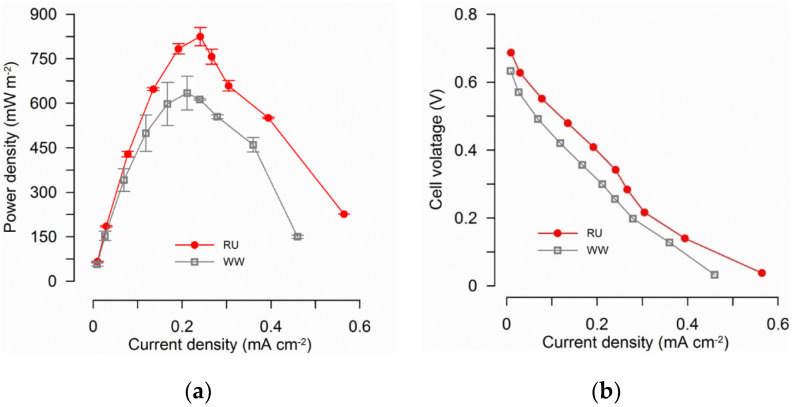
Polarization results for two batch cycles in duplicate reactors for each condition (during the test, external resistance was changed from 10 Ω to 40 kΩ). (**a**) Rumen reactors showed higher power production than other conditions. (**b**) Polarization curves for the two tested conditions showed a reduction in activation losses for rumen reactors, observed by a decrease in the slope at low current densities. RU: rumen plus wastewater; WW: wastewater.

Maximum power densities determined from polarization tests were 824.5 ± 30.8 mWm^−2^ for RU and 634.1 ± 56.4 mWm^−2^ for WW reactors (Figure 2a). Statistical analysis of the three higher values in the power density curves (Figure 2a) shows a significant difference among RU and WW reactors. Polarization curves show that internal resistances were similar for both tested conditions: RU = 201 Ω and WW = 200 Ω (estimated as the slope of the lines in Figure 2b over the current density range of 0.01 to 0.4 mA cm^−2^). The OCP was estimated to be 0.68 V for RU and 0.61 V for WW reactors, based on the Y intercepts of the polarization curves [42].

**Figure 3:**
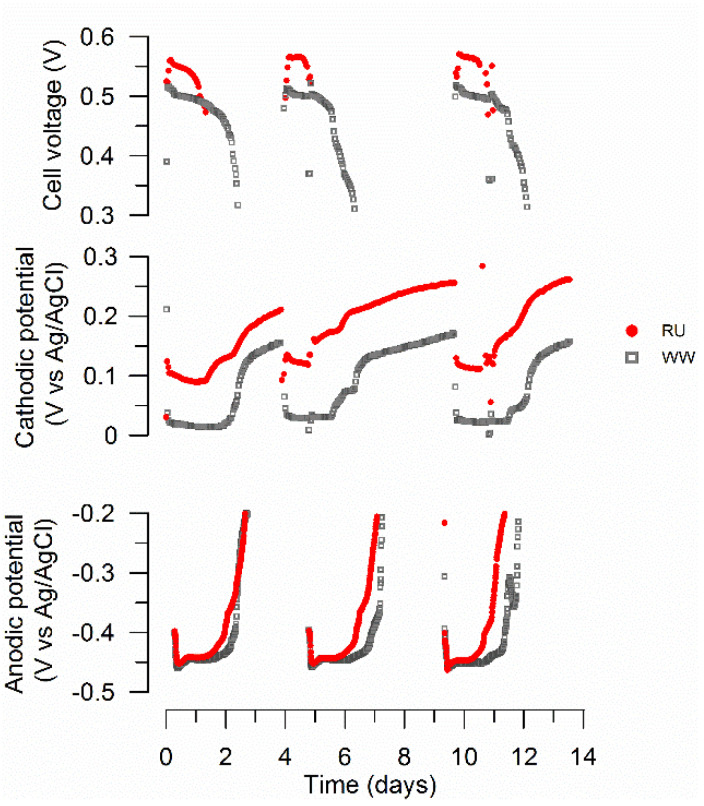
Differences in cathodic potential explain improvements in cell performance in rumen reactors. Cell and cathodic potential for cycles 13, 14, and 15. Anodic potential for cycles 11, 12, and 13. RU: rumen plus wastewater; WW: wastewater. The reactors were connected to external resistance of 1 kΩ.

Increases in cell voltage by adding a rumen inoculum were due to the cathodic potential. The anodic potential measurements did not show significant differences between the two inocula (Figure 3). During each cycle, the air-cathode potentials in the rumen systems ranged from about 0.12 V to 0.26 V (vs. Ag/AgCl, throughout), while in the WW systems, the cathode potentials were between 0.03 V and 0.16 V.

Previous rumen-MFC studies conducted using cellulose as electron donor [14,16] show the presence of electrochemically active rumen bacteria. Using cyclic voltammetry (CV), Zang et al. [16] demonstrated that the electron transfer used by bacteria in an MFC inoculated with rumen fluid and fed with an aquatic plant rich in cellulose (canna) as electron donor was mainly through electron shuttles produced in canna degradation. Increases in voltage observed in this previous study were in the range of days [16], suggesting the occurrence of an indirect electron transfer mechanism. On the other hand, Kiely et al. [25] reported voltage production in about 20 min after medium replacement, suggesting direct electron transfer by bacteria attached to the electrode and no mediators involved in power production. The swift increases in cell potential observed in the present study (Figure 1), and no reductive peaks detected by CV (data not shown), suggest that direct electron transfer was the main mechanism controlling the observed differences in cathode performance. As mentioned earlier, before the MFC inoculation, the rumen fluid was enriched for two weeks in a flask using H_2(g)_ and fumarate as electron donor and acceptor, respectively. Under these conditions, EAB, as *Geobacter sulfurreducens*, may have grown at the cathode, using the electrode instead of hydrogen gas as electron donor [43], enhancing electron flow and current. With this mechanism, microbial activity at the cathode could reduce activation losses and increase power production.

**Figure 4:**
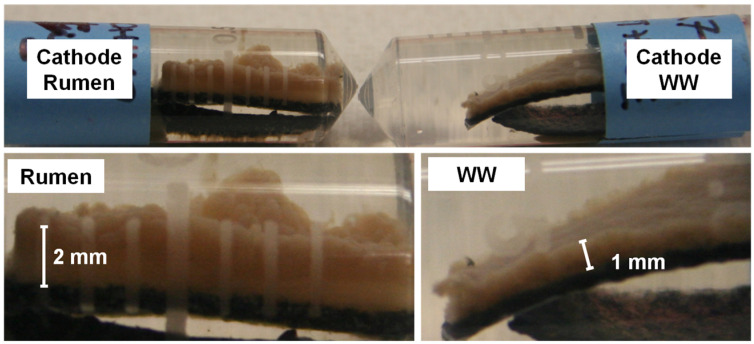
Photographs of biofilm formed on air-cathodes after over 60 days of operation. Bottom panels are a magnification of the top panel (WW: wastewater).

The thickness of these formed air-cathode biofilms was approximately 2 mm for RU and 1 mm for WW (Figure 4). Although the thickness of the RU cathodic biofilm was twice that of the WW cathodic biofilm, the rumen-inoculated systems showed higher cathodic potential and electrochemical activity (presented in the next section). Differences in RU and WW MFC performance suggest that the rumen inoculum yielded cathodic biofilm with increased substrate utilization (i.e., organics removal) and enhanced electron transfer by EAB. Similarly, Chung et al. [44] observed a gradual increase in power density while the biofilm was developed, and a decrease in power when it was removed. This suggests that the cathodic rumen biofilm could facilitate cathodic reactions, resulting in better reactor performance.

**Figure 5:**
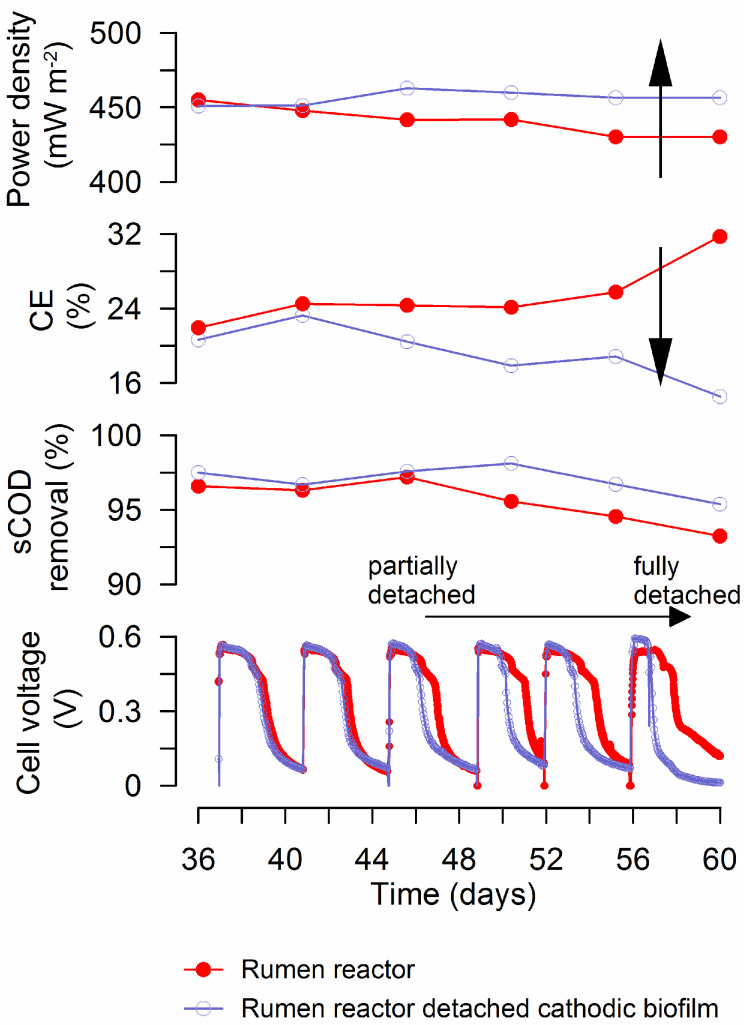
Effect of cathodic biofilm detachment on rumen MFC performance. Loss of the outer part of the cathodic biofilm caused decreases in cell voltages and CE, and slight increases in maximum power densities. Arrows indicate observed trends for Rumen reactors after cathode detachment.

**Figure 6:**
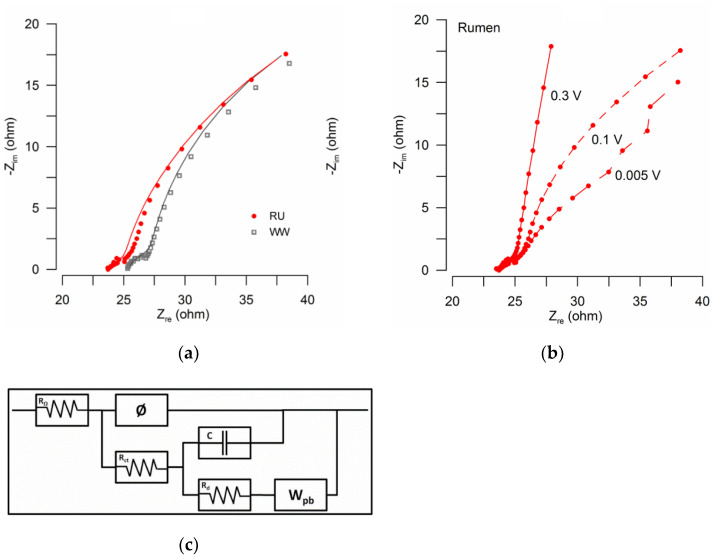
EIS of cathodes. (**a**) Nyquist plot of cathodes tested at 0.1 V. Experimental data (dots) and equivalent circuit model (lines). RU: rumen plus wastewater; WW: wastewater. (**b**) Nyquist plot of rumen reactor cathode poised at different potentials to show the dominance of Rd in the impedance. (**c**) The equivalent circuit was used to fit experimental data. Ohmic resistance (RΩ); Charge transfer resistance (Rct); Diffusion resistance (Rd); Constant phase element (Ø); Capacitor (C); and Porous bounded Warburg element (Wpb).

Additionally, the results of microbial community analysis obtained by Kiely et al. [25] in a study of cathode performance in MFCs fed with different fermentation by-products and Parameswaran et al. [45] in a microbial electrolysis cell study suggest a syntrophic relationship between fermenters such as *Pelobacter* sp. and the known EAB *Geobacter* sp. in reactors fed with fermentable substrates. If present, EAB might be expected to be attached to the electrode (inner part of the biofilm), whereas aerobic and anaerobic bacteria responsible for sCOD degradation might be expected to prefer the outer part of the biofilm with higher sCOD concentrations. Differences in cell voltage between duplicate RU reactors were observed only after 44 days of operation due to a progressive cathodic biofilm detachment in one of the reactors (open diamonds in Figure 5). Interestingly, although a considerable portion of the cathodic biofilm was detached in one RU reactor and a significant decrease in CE was measured (32 to 15%), the voltage peak did not decrease. Even more, a slight increase in power densities was observed (Figure 5), this may be because the thick biofilm impaired proton or hydroxide diffusion (Figure 4 and Figure 6). This presumably non-electrochemically active aerobic and anaerobic consortium, formed in the outer layers of the biofilm, seemed to play an essential role in the degradation of glucose to by-products used by EAB to generate power.

### 3.2. Analysis of Electrochemical Impedances

EIS was performed on anodes and cathodes to quantify and characterize differences in the internal resistance of MFC reactors, with particular interest in the electron transfer resistance in cathodes enriched with the rumen inoculum. EIS analysis of the brush anodes tested at OCP showed ohmic resistances of 16 Ω and 6 Ω for RU and WW, respectively. This might explain the observed slight differences in anodic potentials between these systems (Figure 3). To account for the role of the rumen bio-cathode on power production increases, cathodic EIS was performed at a poised potential of 0.1 V, estimated as the operational cathodic potential at a high current (Figure 3). EIS data were analyzed by fitting an equivalent circuit model (Figure 6) that included ohmic (R_Ω_), charge transfer (R_ct_), and diffusion resistance (R_d_) based on a constant phase element (CPE) model with a double layer charging feature of porous electrodes [46], in this case, the electrochemical properties of the biocathode. To account for mass transfer, the second arc was modeled by adding a capacitor (C) and a porous bounded Warburg (W_pb_) element [47]. The equivalent circuit used was an adaptation of a bioanode model previously used by Jung et al. (2011) [48]. Data were successfully fitted, obtaining goodness of fit (expressed by χ^2^) from 10^−5^ to 10^−3^ [49] (Table 2).

Based on the equivalent circuit analysis, rumen reactors had lower R_Ω_ and R_ct_ than the wastewater inoculum. Additionally, as expected due to the high thickness of cathodic biofilms (Figure 5), for both conditions R_d_ was a dominant factor of the internal resistance due to cathodic biofilm development and the resultant effect on the transport of protons in solution. Interestingly, even though the RU cathode was thicker than the WW cathode, R_d_ values were slightly lower for the rumen reactor. At high potential, diffusion resistance dominated the overall impedance. Figure 6b shows a Nyquist plot of the RU cathode poised at three different potentials (0.005 V, 0.1 V, and 0.3 V), showing the dominance of R_d_ over the impedance. Thus, results of EIS analysis suggested that increases in power production by the rumen inoculum were related to decreases in charge transfer resistance, potentially due to extracellular electron transfer improvement and diffusion resistance. In an electrochemical study of fuel cell cathodes, Springer et al. [50] reported that impedance arcs increased as the overpotential increased due to the conductivity and mass transport limitation within the catalyst layer [50]. Our results (Figure 6b) showed that for increasing overpotential, R_Ω_ and R_ct_ did not significantly change, but R_d_ progressively became the dominant factor of the internal resistance due to biofilm development.

### 3.3. Bacterial Community Analysis

**Figure 7:**
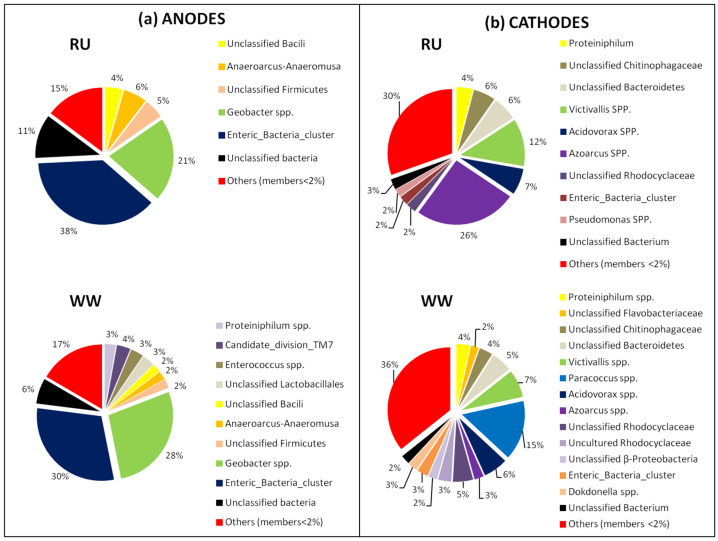
Genus-level community distribution of anodes (**a**) and cathodes (**b**) of rumen (RU) reactors and wastewater (WW).

The dominant anodic community structures were maintained for the RU and WW reactors (Figure 7a), consistent with the nearly identical anodic potentials recorded during operation (Figure 3). Anodic biofilms were dominated by *Firmicutes* (RU = 15%; WW = 13%), *Gammaproteobacteria* (RU = 38%; WW = 30%), and *Deltaproteobacteria* (RU = 21%; WW = 28%). This latter class was predominantly *Geobacter* spp. (RU = 21%; WW = 28%), and there was no significant relative abundance difference of this known electrochemically active group due to inoculation with the rumen enrichment. Previous community characterization in a rumen-MFC study [14] did not report *Geobacter* spp. as members of the anodic community. Hence, *Geobacter* spp. may have been added to the system by the wastewater inoculum.

On the other hand, the pyrosequencing data showed differences in the cathodic biofilm compositions between the RU and WW systems (Figure 7b). The most notable difference in the cathodic communities due to rumen enrichment was the decrease in *Paracoccus* spp. (RU < 2%; WW = 15%) and the increases in *Azoarcus* spp. (RU = 26%; WW = 2%) and *Victivallis* spp. (RU = 12%; WW = 7%) (Figure 7b). The decrease of *Paracoccus* spp. in the reactor inoculated with rumen could have a positive effect on performance. Previous studies had reported electrochemical activity by some species of *Paracoccus* [51,52]. However, in the presence of oxygen and nitrate, *Paracoccus* spp. decrease the availability of compounds used by anodic EAB, negatively affecting the power generation [36]. Observed increases in *Azoarcus* spp. and *Victivallis* spp. relative percentages due to the rumen inoculum could be associated with the observed improvements in substrate degradation (Table 1) and cathode performance (Figure 3 and Table 2). Several studies have reported *Azoarcus* spp. as members of electrode (anode or cathode) microbial communities [6,37,53,54]; however, there are no detailed reports about the electrochemical activity of this group. The second group that showed a significant shift in relative abundance was *Victivallis* spp. (phylum Lentisphaerae). *Victivallis vadensis* has been identified in cow rumen fluids [55] as well as in human gastrointestinal tracts and feces [56]. Recently, *V. vadensis* was identified as a member of an anodic community in a single-chamber MFC [7] and in an anaerobic fluidized bed MFC system [57], suggesting a possible role in electricity production. However, to the best of our knowledge, the electrochemical activity of *Victivallis* spp. has not been reported. *V. vadensis*, a Gram-negative, non-motile, strictly anaerobic bacterium is capable of growth on a range of sugars [56], similar to *Azoarcus* spp, that have been associated with the degradation of various organic compounds using nitrate as an electron acceptor [54]. Hence, the increase in percentages of *Azoarcus* and *Victivallis* spp. that impart high diversity for substrate degradation and electron acceptors other than oxygen, together with the significant increase in the cathodic biofilm thickness (Figure 4), suggests that the rumen enrichment could result in better substrate utilization and changes in biological oxygen consumption at the cathode surface.

Since the rumen fluid was pre-enriched using fumarate as electron acceptor, it was used to test its effects on performance and microbial community distribution. The low percentage of *Paracoccus* spp. observed in Figure 7 for the rumen cathodic biofilm was also observed for rumen reactors amended with fumarate (Figure S1). However, supplementary results show a shift in the frequency of *Victivallis* spp. (from 12 to 20%) and *Azoarcus* spp. (from 26 to 10%) for rumen MFCs operated with fumarate (Figure S1). The effect of fumarate in performance was observed as a decrease in the maximum power density from 824.5 mWm^−2^ exhibited for rumen reactors to 670.3 mWm^−2^ due to fumarate addition, decreasing to the level of the WW reactors (Figure S2).

Additionally, results show a decrease in cell voltage after fumarate addition (Figure S3). These results suggest that fumarate acts as competing electron acceptor, which is concordant with dates reported by Kim and Lee, who showed that *G. sulfurreducens* grows using fumarate as electron acceptor [58]. However, these are contrary to what was observed by Zhang et al. in a pure culture of *Shewanella oneidensis* MR-1, where addition of fumarate to the medium produces an increase in power density, due to fumarate could decrease riboflavin concentration promoting direct electron transfer [59].

Finally, pyrosequencing also revealed changes in cathodic bacterial communities due to rumen enrichment of minor members (denoted as others in Figure 7), including genera that contain known EAB (e.g., *Geobacter*, *Desulfobulbus*, and *Pseudomonas*) and uncultured and unclassified bacteria that may also be playing a role on cathode oxidation. *Pseudomonas* is known to exchange electrons directly with an anode and cathode, which could affect the performance of the reactor [60,61]. Su et al. [61] observed denitrification and dissimilatory nitrate reduction to ammonium by *Pseudomonas alcaliphila* with an electrode as a sole electron donor. Additionally, Cournet et al. [60] reported the electrochemical reduction of oxygen by *Pseudomonas aeruginosa* and *Pseudomonas fluorescens*.

Thus, while the development of a thicker cathodic biofilm was expected to result in higher CE based on the results of other studies, our results also show that it does not necessarily involve decreases in power densities. The presence of syntrophic relationships for substrate degradation and EAB seems to be a key factor to enhance cathodic oxidation, even in the presence of high mass transfer resistance and limitation of proton diffusion from the bulk solution to the catalyst layer due to thicker biofilm formation.

## 4. Conclusions

This study explored, for the first time, the effect of a rumen fluid inoculum on the composition of anodic and cathodic microbial communities in single-chamber air-cathode MFC reactors. The results show that the rumen fluid inoculum increased sCOD removal and power production in the tested MFC reactors. This enhancement was due to the resulted cathodic biofilm, explained by a shift in its microbial composition. Results show an increase in percentages of *Azoarcus* and *Victivallis* species, together with a small occurrence of known EAB, at the rumen inoculated cathode. This research allows us to focus further attention on the effect of new sources of inoculum, such as rumen fluid, on cathodic microbial communities and identify novel bacterial groups that could potentially enhance cathode performance and substrate degradation. Additional efforts are required to investigate the role of *Azoarcus* and *Victivallis* in the cathodic biofilm.

## Figures and Tables

**Table 1:** Average performance observed during approximately 60 days of operation at pH 7, 30 °C, and across 1 kΩ external resistance.

Reactor	sCOD Removal (%)	CH_4_ * (%)	CO_2_ * (%)	CE (%)
Rumen plus WW (RU)	96.1 (± 0.5)	14.9 (± 1.3)	7.1 (± 1.4)	22.8 (± 0.9)
Wastewater (WW)	91.7 (± 4.1)	14.4 (± 2.1)	5.9 (± 1.4)	23.0 (± 0.4)
Rumen plus WW + fumarate (RF)	95.6 (± 0.2)	12.3 (± 1.1)	5.8 (± 0.6)	22.3 (± 1.1)

* Values calculated as the average of 3 batch cycles after 40 days of operation.

**Table 2:** Values of ohmic (R_ohm_), charge transfer (R_ct_), and diffusion (R_d_) resistance calculated by fitting experimental data with an equivalent circuit using χ^2^-minimization.

Parameter	Rumen	WW
R_ohm_ (Ω)	23.9	25.3
R_ct_ (Ω)	3.6	4.1
R_d_ (Ω)	69.2	79.5
Goodness (χ^2^)	1.77 × 10^−4^	2.00 × 10^−4^

## Data Availability

Data sharing is not applicable to this article.

## Supplementary Materials

The following supporting information can be downloaded at: https://www.mdpi.com/article/10.3390/ma15010379/s1, Figure S1: Genus-level community distribution of cathodes of rumen (RU), rumen plus fumarate (RF) and wastewater (WW) reactors, Figure S2: Power density curves of MFC reactors, Figure S3: MFC reactor inoculated with rumen enrichment and amended with 60 mgL^−1^ of fumarate (additional to the 1 gL^−1^ of glucose) for each batch-cycle, during 60 days of operation.
