# Supplementary material for: Rumen Inoculum Enhances Cathode Performance in Single-Chamber Air-Cathode Microbial Fuel Cells
Source: Materials (Basel). 2022 Jan 5;15(1):379. doi: 10.3390/ma15010379 (PMC8746161; doi:10.3390/ma15010379)
Supplement: Supplementary file 1 [file materials-15-00379-s001.zip › materials-1496213-supplementary.pdf]

materials

Supplementary Materials

Rumen Inoculum Enhances Cathode Performance in Single-Chamber Air-Cathode Microbial Fuel Cells

Ignacio T. Vargas [1,2,*], Natalia Tapia [1,2] and John M. Regan [3]

[1] Department of Hydraulic and Environmental Engineering, Pontificia Universidad Católica de Chile, Santiago 7820436, Chile; netapia@uc.cl
[2] Centro de Desarrollo Urbano Sustentable (CEDEUS), Santiago 7520246, Chile
[3] Department of Civil and Environmental Engineering, The Pennsylvania State University, University Park, PA 16801, USA; jmr41@psu.edu
* Correspondence: itvargas@ing.puc.cl

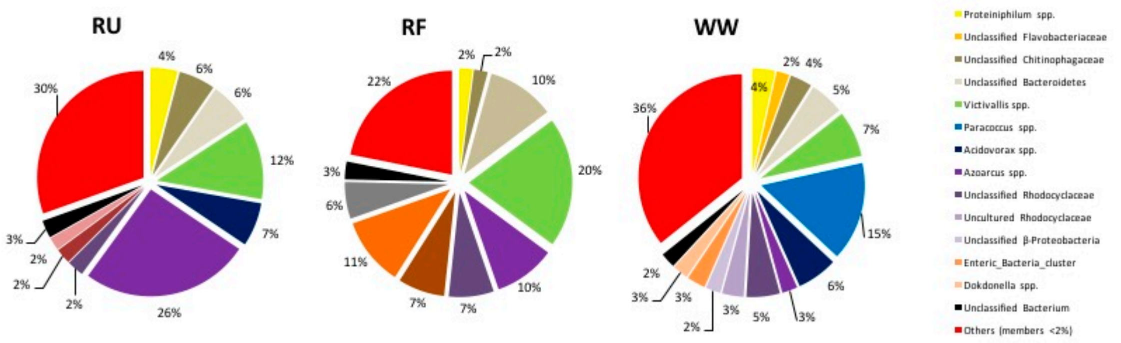

Figure S1. Genus-level community distribution of cathodes of rumen (RU), rumen + fumarate (RF) and wastewater (WW) reactors. Results show a shift in the frequency of *Victivallis* spp. (from 26% to 20%) and *Azoarcus* spp. (from 3% to 10%) for rumen reactors amended with fumarate.

Citation: Vargas, I.T.; Tapia, N.; Regan, J.M. Rumen Inoculum Enhances Cathode Performance in Single-Chamber Air-Cathode Microbial Fuel Cells. *Materials* **2022**, *15*, 379. https://doi.org/10.3390/ma15010379

Academic Editors: Sneha Samal and Ignazio Blanco

Received: 21 November 2021
Accepted: 31 December 2021
Published: 5 January 2022

Publisher's Note: MDPI stays neutral with regard to jurisdictional claims in published maps and institutional affiliations.

Copyright: © 2022 by the authors. Licensee MDPI, Basel, Switzerland. This article is an open access article distributed under the terms and conditions of the Creative Commons Attribution (CC BY) license (https://creativecommons.org/licenses/by/4.0/).

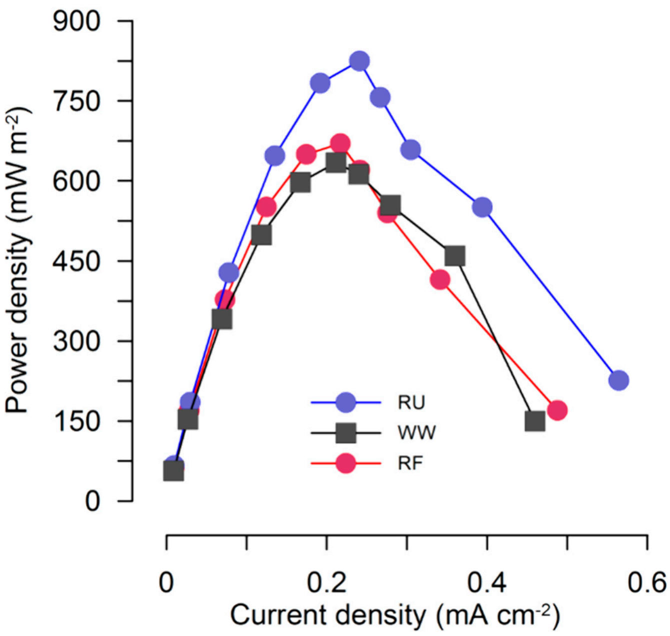

Figure S2. Power density curves of MFC reactors. A decrease in the maximum power density is observed in rumen reactors amended with fumarate. RU: rumen and wastewater inoculum; RF: rumen and wastewater inoculum + fumarate additions; WW: wastewater inoculum.

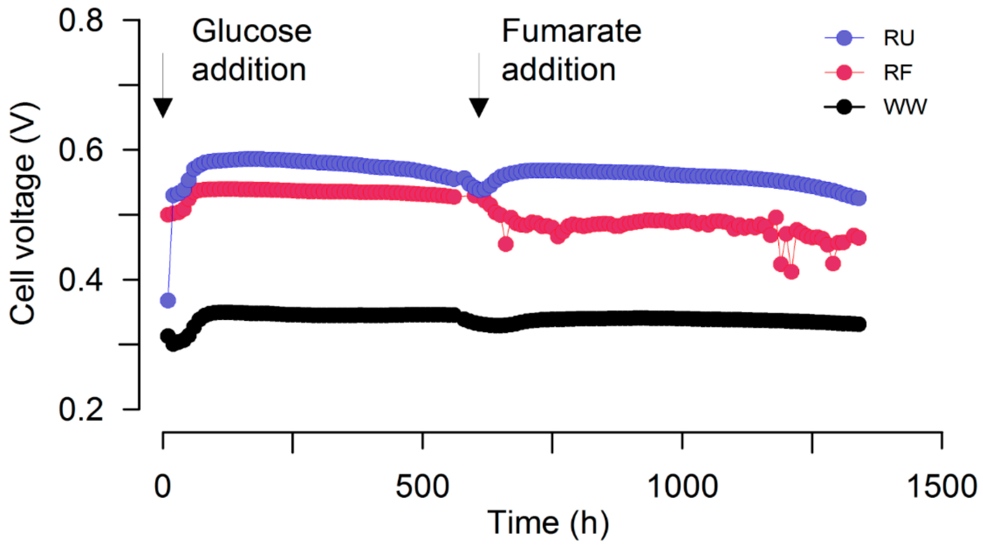

Figure S3. MFC reactor inoculated with rumen enrichment and amended with 60 mgL^{-1} of fumarate (additional to the 1 gL^{-1} of glucose) for each batch-cycle, during 60 days of operation. Results show a decrease in cell voltage after fumarate addition suggesting its role as alternative electron acceptor. RU: rumen + wastewater; RF: rumen + wastewater amended with fumarate; WW: wastewater.
